# The ‘Glasgow effect’: the controversial cultural life of a public health term

**DOI:** 10.1136/medhum-2022-012594

**Published:** 2023-11-30

**Authors:** Fred Spence

**Affiliations:** 1 School of Critical Studies, University of Glasgow, Glasgow, UK

**Keywords:** Public health, Medical humanities, journalism, cultural history, literary studies

## Abstract

The question of why more people in Glasgow were dying, and younger, compared with English cities with almost identical levels of deprivation, was a hot topic in Scottish public health debates in the early 21st century. Public health researchers, particularly the Glasgow Centre of Population Health (GCPH), used the terms ‘Glasgow effect’ and ‘Scottish effect’ as placeholders while identifying the unknown factors behind Scotland’s excess mortality. Yet the terms took on a colourful life of their own in the press and larger culture and continue to circulate, despite GCPH’s attempts to retire them. This paper is the first to analyse the cultural life of the ‘Glasgow effect’ and ‘Scottish effect’ terms. Looking primarily at the Scottish press 1998–2022, I analyse the politically charged and often controversial debates and lay recommendations around the concepts. I also trace the terms’ parallel usage, and indeed origin, in contexts unrelated to health. I argue that the ‘Glasgow effect’ functions as a myth. This myth emphasises Scottish exceptionalism in public health and larger culture, at a time when devolution and the prospect of independence heightened optimism and anxiety about Scotland’s future. It overlaps with a larger and longstanding myth of Scottish cultural pathology, or the pathological Scot. The flexibility of the ‘Glasgow effect’ and ‘Scottish effect’ terms is exploited by journalists, academics and artists to serve competing agendas, establish their own expertise and influence public opinion. While it may now be challenging to eradicate these terms, especially in lay contexts, researchers and policy makers should avoid using these unstable terms uncritically. The example of the ‘Glasgow effect’ shows how health concepts can become wrapped in larger national or political narratives and highlights the difficulties for public health communicators in introducing complex and emerging public health ideas into a dynamic landscape of lay beliefs.

## Introduction

 Why are so many more Glaswegians dying, and younger, compared with Manchester and Liverpool, English cities with almost identical levels of deprivation? This was a hot topic in debates about Scottish public health in the first decades of the 21st century. If deprivation, a common factor behind health inequalities, could not alone account for Glasgow’s excess mortality, then what was the mysterious cause? Public health researchers, particularly the Glasgow Centre for Population Health (GCPH), used the terms ‘Scottish effect’ and ‘Glasgow effect’ to gesture towards these as yet unknown factors. The press was hooked. As an unexplained phenomenon with no immediate solution, the ‘Glasgow effect’ became what GCPH’s David Walsh scathingly called ‘a Scooby-Doo mystery’ (Macdonald 2019, para 3 of 72). Was Scottishness itself to blame, or a lack of daylight, or was there, so to speak, something in the water? These vague but catchy terms took on a colourful life of their own. Their ambiguity left them open to revision and interpretation, a malleability that appealed to a broad range of canny journalists, academics and artists. In the press, the ‘Scottish effect’ and ‘Glasgow effect’ were often misunderstood or misrepresented. Recommendations for addressing the unknown cause(s) of Scotland’s excess mortality typically focused on individuals optimising their own behaviour and emotions. Some suggestions were considerably more provocative and controversial, with discussions often straying into debates about national character or Scotland’s political future. To complicate their definitions further, both terms originated in contexts unrelated to health, on disparate topics such as cultural regeneration and Scottish voting trends, and continued to circulate alongside the public health usage.

This paper is the first to analyse the cultural life of the loaded and complicated terms ‘Scottish effect’ and ‘Glasgow effect’ in the press and larger culture, asking why they became so resonant at this moment in Scottish history. As a literary medical humanities scholar, my focus is not on GCPH’s work, its influence on policy or the sociological reality of excess mortality in Scotland. I am interested in the press reception and cultural life of the ‘Scottish effect’ and ‘Glasgow effect’ terms as they were adopted by actors with various agendas and/or presented to lay audiences, in often contradictory and controversial ways. I conclude by further theorising on the ‘Glasgow effect’ concept, asking where it fits in the intellectual ecology, how it functions and who benefits from its usage. I am interested in these terms as myths, that is, a story that helps define a group or nation to themselves and others (McCrone 2001, 79). The ‘Glasgow effect’ myth gained prominence in the years between Scottish devolution in 1997 and the 2014 Scottish independence referendum. Is the ‘Glasgow effect’ simply an extension of the larger myth of Scottish pathology (discussed below), or does it do something different, arising in this specific cultural moment and relating to a real public health trend?

Studying health in a Scottish context offers rich material for the medical humanities scholar. After all, the country’s poor public health has earned it the reputation of the ‘sick man of Europe’ and texts about mental illness, suicide and alcoholism are prominent in the Scottish canon. Yet questions about nation, identity and voice have tended to dominate Scottish studies, with little dialogue with the medical humanities to date. Similarly, the critical medical humanities could be enriched by closer interaction with Scottish studies. Few, if any, understandings of health and illness are transhistorical or transcultural. As Lisa Diedrich (2007, 61) argues, illness narratives reflect ‘national attitudes and/or ideologies about illness and death’ and help create ‘imagined national communities of the healthy and the ill’. While the national arts of being ill are fictions, they are useful to understand ‘specific cultural narratives of illness’ and ‘particular cultural anxieties’ (xxi). The nuances of the national and the regional risks being lost under the broad categories of Anglo-American or Western. I seek to refresh debates in both fields by bringing them into dialogue.

### Defining the ‘Glasgow effect’

In 2010, GCPH published a report titled ‘Investigating a “Glasgow Effect”’. It explains that ‘Scottish effect’ describes ‘the higher levels of mortality and poor health experienced in Scotland over and above that explained by socio-economic circumstances’. The term ‘Glasgow effect’ then followed in light of evidence that this excess is concentrated in West Central Scotland (Glasgow Centre for Population Health 2010, 7). The term describes the ‘poor health status of Glasgow *over and above* that attributable to the city’s high levels of socio-economic deprivation’ (original emphasis) (Glasgow Centre for Population Health 2010, 4). Compared with Liverpool and Manchester, post-industrial English cities with almost identical deprivation profiles, Glasgow deaths were 15% higher, while premature deaths (those before 65) were 30% higher (8). This excess was ‘seen across virtually the whole population: all ages (except the very young), both males and females, in deprived and non-deprived neighbourhoods’. GCPH concluded that deprivation, a key determinant of health, could not alone explain Glasgow’s higher levels of mortality (Glasgow Centre for Population Health 2010, 8). The term ‘Glasgow effect’ was a placeholder, gesturing towards the unknown factors behind this excess mortality until these factors could be identified. GCPH’s 2010 report outlines varied hypotheses, from societal or family breakdown and genetic factors, to the effects of migration and differences in spatial patterning of deprivation (Glasgow Centre for Population Health 2010, 47).

A follow-up report by GCPH in 2016 identified the previously unknown factors. It emphasises that much of Glasgow and Scotland’s poor public health is indeed explained by deindustrialisation, deprivation and poverty, the root causes of poor health in many modern societies (Glasgow Centre for Population Health 2016, 7). Since the late 1970s, social and economic policies produced widening socioeconomic and health inequalities across the UK. Glasgow was simply more vulnerable to this, resulting in the city’s excess mortality (Glasgow Centre for Population Health 2016, 8). There are a variety of reasons for this vulnerability, such as high levels of deprivation historically, the prioritisation of gentrification and commercial development by Glasgow’s local government, and the socially selective New Town programme, which redirected industry, investment and young, skilled workers away from Glasgow and towards newly-built towns outside of the city (Glasgow Centre for Population Health 2016, 8-9). The comparator cities may have also had protective factors: in Liverpool, higher levels of social capital, such as community ties and participation in politics; in Manchester, greater ethnic diversity and the associated ‘healthy migrant effects’. A ‘democratic deficit’ in Glasgow, characterised by ‘feelings of despondency, disempowerment, and lack of sense of control’, is also a psychosocial risk factor (Glasgow Centre for Population Health 2016, 9). Lastly, GCPH highlight that the measurement of poverty and deprivation may be inadequate (Glasgow Centre for Population Health 2016, 9) and acknowledge a number of smaller, unnamed additional factors (Glasgow Centre for Population Health 2016, 10). The mystery of Glasgow’s poor health proved not so mysterious after all, with deindustrialisation, deprivation and poverty unsurprisingly to blame.

The year of the 2016 follow-up report, GCPH publicly distanced themselves from the term ‘Glasgow effect’, arguing for its retirement and contesting what it saw as distortion of its concepts and findings in the media. GCPH’s public health programme manager David Walsh published a blog post on the GCPH website titled ‘The “Glasgow Effect” and the “Scottish Effect”: unhelpful terms which have now lost their meaning’. Walsh (2016, para 9 of 13) argues that the terms are now redundant as the excess mortality is no longer unexplained. He is also critical of their usage in the press, noting that ‘Some journalists loved these expressions: the idea of an unexplained mystery was (and still is) lapped up by some in the media’. Lots of unlikely explanations were proposed, such as Scotland’s excess mortality being ‘caused by the weather, or by deep fried mars bars’ (Walsh 2016, para 5 of 13). He complains that the terms were used in ‘all sorts of different and irrelevant contexts’, unrelated to health, which ‘muddied their meaning’. The terms were also misused as explanations in themselves, as in the claim that people die younger *because* of the ‘Glasgow effect’ (Walsh 2016, para 6 of 13). For these reasons, Walsh explains, GCPH retired the terms several years previously, preferring ‘excess mortality’ (Walsh 2016, para 8 of 13). However, ‘Glasgow effect’ had already taken on a colourful life of its own in the press, policy and academia and continues to circulate. GCPH lost control of the term but, as I will show, the organisation never really had full ownership. Indeed, both ‘Scottish effect’ and ‘Glasgow effect’ were already circulating in the press before GCPH claimed them as their own.

## Methodology

I am interested in the cultural life of the ‘Scottish effect’ and ‘Glasgow effect’ terms, particularly their usage in the press, in media made by and for lay audiences. Newspaper articles are worth studying because, as Lupton (1992, 148) highlights, the press plays an important role in setting the agenda around health issues. Lupton (1992, 148) argues that discourse analysis is useful to understand the formulation and expression of lay health beliefs in the press and other media. While quantitative content analysis approaches a text descriptively, counting instances of a phrase or theme, discourse analysis looks critically at language (Lupton 1992, 145). It reveals subtext beneath the obvious surface meaning, exploring how language reflects and perpetuates dominant ideologies and power structures (Lupton 1992, 147). I approach my texts as a literary scholar, attuned to these nuances and subtexts. Using the Lexis Library News database, I analysed four newspapers: Scotland broadsheet *The Herald*, UK broadsheet *The Guardian*, Scotland tabloid *The Daily Record*, and UK tabloid *The Sun*. These newspapers were chosen to determine if different attitudes about Scottish public health were present in broadsheets and tabloids, or newspapers published within and outside Scotland. As Christine Knight (2016, 374) highlights, the treatment of Scotland in London-based UK media can differ from indigenous newspapers or Scottish editions of national publications. Although I focus on these four newspapers, I occasionally draw on other press and academic publications to uncover a fuller history of the terms.

The majority of articles about the ‘Glasgow effect’ or ‘Scottish effect’ are in *The Herald*. Of the 16 articles selected using the term ‘Scottish effect’, 12 come from *The Herald. The Daily Record* and *The Guardian* each have two articles, with no articles found in *The Sun*. Of the 64 selected results for ‘Glasgow effect’, half of these, 32 articles, come from *The Herald*. Seventeen articles are published in *The Guardian*, 12 in *The Daily Record* and 3 in *The Sun*. However, many of these articles use the term ‘Glasgow effect’ outside of public health contexts to discuss cultural achievements or arts projects, as I discuss below. A reader of *The Herald*, *The Guardian* or *The Daily Record*, then, would find several competing meanings of ‘Glasgow effect’ within the same newspaper. None of *The Sun* articles discuss public health. The broadsheets studied here, especially *The Herald*, are significantly more invested in these public health concepts than the tabloids. Even when discussing health, *The Herald* uses a broad range of tones and perspectives, from straightforward reporting to irreverent articles and speculative, strongly worded opinion pieces.

### Origins of ‘Scottish effect’ and ‘Glasgow effect’

‘Scottish effect’ and ‘Glasgow effect’ are mobile terms with complicated histories in the press. Simon D.S. Fraser and Steve George explain the origins of the health term ‘Scottish effect’. In 1989, Vera Carstairs and Russell Morris (of the Carstairs deprivation index) compared mortality in Scotland, England and Wales. They suggested that Scotland’s excess mortality might be explained by greater deprivation but could not demonstrate a causal relationship. The ‘Scottish effect’ concept emerged to describe Scotland’s excess of poor health beyond what could be explained by common variations, like differences in age distribution (Fraser and George 2015, 100). A publication called ‘The Scottish Effect?’ was produced by the Scottish Council Foundation’s Healthy Public Policy Network in 1998 (Healthy Public Policy Network 1998b). The term first appeared in newspapers that year in a *Herald* article by Phil Hanlon, a member of the Healthy Public Policy Network and later a key figure in GCPH’s work. Hanlon’s article discusses another 1998 report by the Scottish Council Foundation called ‘The Possible Scot’ (Healthy Public Policy Network 1998a). He highlights that while deprivation is central to health problems in both countries, there has been a striking growth in Scottish mortality compared with England. Hanlon questions whether there is a ‘Scottish effect’, ‘additional and distinctive factors’ peculiar to Scotland’s ‘culture or ecology’ (Hanlon 1998, para 3 of 8). ‘Glasgow effect’ is the more commonly used term in the press. Its public health meaning was introduced in *The Herald* in 2004. The article explains that the GCPH has been launched to ‘address Glasgow’s poor health record’ and, according to health minister Andy Kerr, will ‘examine how attributes like sense of control, optimism and confidence impact on determining health outcomes’ (Puttick 2004, para 1 and para 11 of 11). GCPH manager Professor Carol Tannahill is quoted saying ‘[w]e believe there’s something unique to the city that we’re calling the “Glasgow effect”’ (para 6 of 11). There is no further definition of ‘Glasgow effect’ given, beyond Tannahill’s observation that the reasons behind Glasgow’s poor health are ‘more complex’ than poverty and deprivation (para 8 of 11).

However, the terms ‘Scottish effect’ and ‘Glasgow effect’, with different meanings unrelated to public health, were already circulating in the press. Ten years before Hanlon’s *Herald* article, ‘Scottish effect’ appears in the headline of a 1987 *Times* article: ‘The Queen’s Speech—Reaction: Ministers moves to allay “Scottish effect” in London’ (Warman 1987). The article discusses Conservative backbench opposition to Margaret Thatcher’s controversial new ‘community charge’, more commonly known as the ‘poll tax’. Replacing property rates, which only two-fifths of the electorate paid, the poll tax applied a flat rate to every adult (Lamb 2020, 458). Opposition was widespread throughout Britain but particularly so in Scotland, where the poll tax came into effect a year before England and Wales, in 1989 (459). Probably in part due to the poll tax, the Conservatives’ vote share in Scotland declined significantly in the 1987 general election, despite the party winning a comfortable UK majority overall (Denver 1987, 449–50). The *Times* article claims that London MPs are concerned about the damage the poll tax might cause the Government and the replication of this ‘Scottish effect’ of poor election results in London. This usage appears again in a 1992 *Guardian* article on that year’s general election. The article predicts a decline of Conservative seats in certain English regions: this will be ‘the beginning of the Scotland effect [sic]; but in England’s North-west’ (‘North by North-West—A Nailbiting Cliffhanger for the Tories’, The Guardian 1992, para 7 of 9). ‘Scottish effect’ continues to circulate, although infrequently and with a variety of additional meanings. Almost three decades after the Conservatives’ poor election results in 1987, ‘Scottish effect’ is used in *The Herald* to describe fears of Scotland’s disproportionate influence on the 2015 general election (Wollard 2015, para 7 of 10). Meanwhile, *The Belfast Telegraph* in 2014 suggests that Northern Irish interest in a referendum on Irish unity may be ‘boosted by a Scottish effect’ with the influence of Scotland’s own independence referendum that year (Clarke 2014, para 2 of 7). The term is also used outside of political contexts. An article from 2001 in *The Sunday Telegraph* recommends that English urbanites looking for a second home consider rural Scotland because of Scotland’s relatively inexpensive property prices: ‘even a stone’s throw from the big cities, the “Scottish effect” can prevail, keeping prices low’ (West 2001, para 13 of 19). While the public health meaning of ‘Scottish effect’ has persisted and predominated, these ephemeral alternative meanings make the term more complicated and unstable. ‘Scottish effect’ is a phrase recognisable enough that it keeps returning but vague enough that it is redefined each time.

‘Glasgow effect’ is the more commonly used term in the press. It too circulates with a variety of meanings, related to cultural regeneration and achievement. Glasgow won the European Capital of Culture award in 1990. This cultural investment regenerated the city, a striking transformation that *The Guardian* in 2002 called ‘the 1990 effect’ and ‘the Glasgow effect’, following ‘the Guggenheim effect’ (Ward 2002, para 6 and para 12 of 20). The ‘Guggenheim effect’ describes the similar regeneration of Bilbao, a Basque city in industrial decline, after the 1997 opening of the Guggenheim Museum; the building’s innovative design attracted unprecedented numbers of tourists to the area. According to Plaza, Tironi, and Haarich (2009, 1711), this captured attention from policy-makers worldwide. In the early 2000s, UK cities like Liverpool, Oxford and Cardiff competed for the European Capital of Culture title. The ‘Glasgow effect’ is a major part of this coverage, especially in *The Guardian*, and a key factor in the title’s desirability. Sir Bob Scott, Liverpool’s bid leader, boasts that Liverpool has the strongest application because it is ‘most likely to replicate the “Glasgow effect”’ (Carter 2003, para 12 of 13). This cultural regeneration meaning of ‘Glasgow effect’ circulated in the national press in 2002 and 2003; in 2004, Tannahill introduced its public health meaning. Even if GCPH based ‘Glasgow effect’ on ‘Scottish effect’, the public may have been familiar with this original cultural regeneration meaning.

In the following years, public health meanings of ‘Glasgow effect’ dominate, yet usage related to Glaswegian culture persists across a range of publications. *The Scottish Sun* in 2013 describes Glasgow-based Father Sculptor as ‘the latest band to benefit from the Glasgow effect’, comparing them to successful Scottish bands Franz Ferdinand and Snow Patrol. The city is ‘a fertile breeding ground for music’, conferring a distinctive artistic edge (Gellatly 2013, para 1–2 of 9). In a 2014 *Herald* article, Glasgow Film Festival’s co-director Allison Gardner attributes the festival’s success to its enthusiastic public audiences, explaining that distributors ‘love what is being called the “Glasgow effect”, they love gauging the reactions of the audiences, and it is the audiences that have made this festival’ (Miller 2014, para 15 of 24). This suggests that ‘Glasgow effect’ has a wider usage outside of journalism and Scotland, separate from its public health meaning. The term is also used positively in a sporting context in *The Guardian*, as with the success of rugby team the Glasgow Warriors: ‘the Ireland coach hopes Scotland will be inspired by the Glasgow effect—Gregor Townsend’s Warriors are the Celtic club champions’ (Averis 2015, para 8 of 15). ‘Glasgow effect’, then, always had multiple meanings beyond public health. A newspaper reader in Scotland may have encountered the ‘Glasgow effect’ as a desirable signifier of regeneration, a marker for unexplained health inequalities, and as praise for Glaswegian cultural and athletic success—sometimes simultaneously and all in the space of a decade. ‘Glasgow effect’ is a flexible, even contradictory term, oscillating between positive and negative, cultural and health meanings in public discourse.

The most (in)famous non-public health use of ‘Glasgow effect’ was as the title of a 2016 durational art project by Harrison (2016), funded by Creative Scotland. ‘Part psychological experiment, part protest, part strike’, ‘The Glasgow Effect’ involved Harrison staying within Glasgow’s city limits for a year and avoiding all vehicles except her bicycle, reducing her carbon footprint for transport to zero (Harrison 2019, 17). Harrison aimed to highlight ‘the relationship between literal and social mobility, between class and carbon footprint’ (Harrison 2019, 141) and the interconnections between ‘social, environmental and economic injustices’ (17). The project’s aims were lost amid online controversy after Harrison posted an image of chips on the project’s Facebook page, a marker for the city’s stereotypically poor diet. Many were outraged that Harrison received public money, through Creative Scotland, to stay in Glasgow, while poorer residents could not choose to leave. The criticism in the press and online focused nearly exclusively on class and England-born Harrison’s perceived status as a privileged outsider. Newspaper coverage of this controversy alters its definition of the ‘Glasgow effect’ to feed into this class discussion. In *The Daily Record*, the ‘Glasgow effect’ is defined as ‘poor life expectancy of working-class Glaswegians’ (Kerr 2016, para 5 of 23) and, in *The Herald*, as ‘poor health and low life expectancy’ in Glasgow’s ‘most deprived areas’ (Miller 2016, para 13 of 21). This redefinition is surprising in *The Herald*’s case as it had covered the ‘Glasgow effect’ for years. The term is evidently flexible enough to be redefined when convenient. While Walsh complains that the apparent misuse of ‘Glasgow effect’ muddied its public health meaning, this polysemy was always present with both terms. ‘Glasgow effect’ and ‘Scottish effect’ could never have had the single public health meaning that GCPH desired.

### The ‘Glasgow effect’ and public health discussions

Despite these alternative meanings, ‘Scottish effect’ and ‘Glasgow effect’ are most commonly used in relation to public health in the press. Typically they are associated with ‘soaring suicide rates’ and ‘heavy drinking’ (Stewart 2012, para 11 of 28) as well as ‘high rates of mental illness’, poor diet and limited exercise (Herald 2009, para 6–7 of 14). Yet even within this context, the term is very flexible and invites striking arguments, in both newspaper and academic articles. In the press, public health definitions of ‘Scottish effect’ and ‘Glasgow effect’ are occasionally distorted from GCPH’s definition. The suggestion that the ‘effects’ themselves are responsible for poor health is a key way in which the terms are misrepresented. *The Herald* in 2010 claims that research ‘points to a Glasgow effect or Scottish effect which results in early death for adults in the West of Scotland’ (Herald 2010, para 6 of 9). This is, then, an effect which results in itself. Similarly, *The Guardian* in 2016 describes Govan resident Jean Melvin as, at 92, ‘an exception to the Glasgow effect rule’, as if early death in Glasgow is inevitable rather than just statistically more likely (Goodwin 2016, para 20 of 57). ‘Glasgow effect’ is sometimes redefined, whether intentionally or not, to better suit the context or argument. Writing about a community garden group, *The Daily Record* defines the ‘Glasgow effect’ as ‘poor diet and lack of activity combining to shorten life expectancy’ (English 2014, para 18 of 28). Gerry Hassan (2010, para 7 of 14), commenting on the Scottish political classes, claims that the ‘Glasgow effect’ ‘shows [that] allowing for the socio-economic makeup of the city, [Glasgow] is significantly worse in terms of poverty, crime and violence’, without reference to public health. These redefinitions create more uncertainty around the term, allowing for more flexible usage.

As unexplained phenomena, there was no clear or immediate solution to the ‘Scottish effect’ and ‘Glasgow effect’, creating something of a vacuum. Even after GCPH identified the factors behind Glasgow’s excess mortality and retired the term in 2016, ‘Glasgow effect’ continues to circulate in the press, described as a ‘mystery’ (Allan 2017, para 2 of 10), ‘impenetrable’ (Mclaughlin 2018, para 2 of 14) and ‘an enduring public health puzzle’ (McArdle 2018, para 1 of 38). Philosopher Nancy Tuana (2004) argues that ignorance is often not a simple knowledge gap but an ‘active production’, linked to ‘cognitive authority, doubt, trust, silencing, and uncertainty’. The press maintained ignorance around the ‘Glasgow effect’. This allowed journalists to change the conversation from structural inequality and policy, as was GCPH’s focus, to culture and personal responsibility. Lifestyle change is the default recommendation, focusing on alcohol, diet, exercise, confidence and optimism. Although the link between poverty and poorer health is well established, *The Herald* suggests that addressing poverty is irrelevant as Glasgow’s excess mortality goes beyond class boundaries. With ‘the odds stacked against us’ due to ‘a mysterious Scottish factor’, ‘we must make an extra effort to improve our health’ as individuals, by reducing consumption of alcohol, drugs and fatty, sugary foods (‘Health warning that must not be ignored’ 2010, para 8–9 of 9). The ‘Scottish effect’ and ‘Glasgow effect’ concepts seem to shut down the possibility of effective structural intervention. The focus on individual lifestyle chimes with neoliberal common sense. The cultivation of ignorance around Glasgow’s health inequalities entrenches neoliberal ideas about self-regulation and diminished state responsibility.

This trend of individualising health issues is common well beyond Scotland. These lifestyle-focused recommendations exemplify what Jennie Popay *et al* call ‘lifestyle drift’. This is the tendency for policy to recognise the need for structural solutions to health inequalities, only to ‘drift’ to focusing on individual lifestyle factors. Action to address structural issues is then neglected or undermined (Popay, Whitehead, and Hunter 2010, 148). The focus on lifestyle in ‘Glasgow effect’ discussions prescribes a kind of inverted quarantine. Sociologist Andrew Szasz (2007, 2-3) defines inverted quarantine as ‘individualised acts of self-protection’ to avoid toxins and contaminants. When dealing with environmental risk factors, individuals now act as consumers, buying bottled water or organic food, rather than acting as political citizens and advocating for environmental policy changes. The basic dyad in quarantine, ‘healthy overall conditions/diseased individuals’, is inverted to ‘diseased conditions / healthy individuals’. In this formation, the environment induces illness; the threat cannot be contained in a discrete location or individual. Healthy individuals protect themselves by withdrawing behind a (figurative) barrier, such as drinking bottled rather than tap water (Szasz 2007, 5). This inverted quarantine, Szasz argues, causes ‘political anesthesia’. When individuals feel they are protecting themselves, they are less motivated to pursue collective change for collective benefit (Szasz 2007, 195). A kind of inverted quarantine is often invoked in ‘Glasgow effect’ discussions. Scotland, as place or culture, is imagined as the ‘diseased conditions’. Healthy individuals must protect themselves through the barrier of lifestyle changes, such as drinking less alcohol or becoming more optimistic. Following Szasz’s argument, this may also produce political anaesthesia about the socioeconomic circumstances behind many health inequalities.

The single-minded focus on individual lifestyle leads to some idiosyncratic and colourful arguments. In *The Herald*, Colette Douglas Home discusses the theory that the ‘Scottish effect’ is related to low levels of sunlight. Home urges readers to take vitamin D supplements despite acknowledging that this has not been recommended by the Chief Medical Officer (Home 2011, para 7–9 of 31). While Home writes that ‘poverty and social isolation lie at the root of Scotland’s rotten health’ (para 19 of 31), she insists that ‘[w]e are free to be the masters of our own destiny’. That is, ‘[w]e can smoke, eat damaging foods[,] drink ourselves to death’ and ‘starve our bodies’ of vitamin D, or ‘we can make sure our [vitamin D] levels remain high’ through supplements (Home 2011, para 28 of 31). The word ‘starve’ implies that not taking supplements is negligent. It is revealing that Home also attacks smoking, drinking and diet, unrelated to supplements. Home ends with a striking image, drawing on evolutionary psychology: ‘Our ancestors didn’t survive by waiting for the appointed monkey to tell them when to jump from the jaws of a tiger. And I’ll not cross my fingers that I stay healthy while I wait for the state to complete its research and act as nanny. I hope you don’t either’ (Home 2011, para 31 of 31). The monkey analogy suggests that it is foolish and dangerous to passively wait for state instructions. Health must be actively cultivated. While Home insists on trusting natural instinct, her chiding tone is intended to goad readers into compliance, despite the lack of evidence on the safety and effectiveness of supplements. Home repeatedly uses ‘we’, a common rhetorical strategy in ‘Scottish effect’ and ‘Glasgow effect’ articles, implying collective experience while advocating individual change.

Colourful arguments also appear in academic writing. In a *Journal of Bioethical Inquiry* editorial, David M. Shaw (2015, 11) argues that since the unknown factors behind the ‘Glasgow effect’ cannot be addressed, Glasgow parents may have ‘an ethical obligation’ to relocate their children. He uses the analogy of a ‘weak “dirty bomb”’: many would leave if background radiation increased the risk of early mortality by more than 15 per cent, the level represented by the ‘Glasgow effect’ (Shaw 2015, 12). This analogy and the focus on geography in the absence of known factors invites interesting parallels to miasma theory. Darby Wood Walters (2019) explains that miasma theory, which reached peak popularity during the mid-nineteenth century, was uncertain about the origins of disease and envisioned miasma as ‘a shapeless and undetectable property of air in certain locations’. Perhaps Shaw imagines a Glasgow miasma. Hassan (2016, para 8 of 22) in *The Daily Record* writes that the ‘Glasgow effect’ has ‘come to mean something intangible, almost if not in the air, then the culture, which is all-powerful and cannot be resisted’. Miasma is a powerful image for representing something as intangible as poorly understood health inequalities. The focus on diet and alcohol in ‘Glasgow effect’ coverage is today medically sanctioned common sense. Yet it may also contain residues of miasmatic thinking, in the anxiety over controlling harmful contaminants entering the body. Shaw illustrates his relocation proposal through the anecdotal Gavin and Esther, a young couple with children who, although tirelessly health-conscious and well-behaved, are ‘victims of this strange, unexplained effect’ (Shaw 2015, 12). This phrasing gives the ‘Glasgow effect’ malicious agency. Walters (2019, 598) explains that Jack the Ripper was associated with miasma in press coverage because both were frighteningly unknowable and unexplainable. Shaw draws this same connection between murder and disease, recasting the ‘Glasgow effect’ as a monstrous serial killer. The thrill of an unknown predator may partly explain the term’s continued resonance. Whether Shaw earnestly believes his own argument or is capitalising on a hot topic, ‘Scottish effect’ and ‘Glasgow effect’ prove to be flexible, provocative concepts, in both popular and academic writing.

GCPH thoroughly tested and rejected these popular hypotheses. Walsh *et al*. (2014, 2) compared Glasgow, Liverpool and Manchester on a ‘sense of coherence’ scale. This assesses how people manage stress to protect their health, asking whether life feels comprehensible, manageable and meaningful. The Glasgow sample’s ‘sense of coherence’ was substantially higher, not lower, compared with Liverpool and Manchester (3), and thus an unlikely explanation for Glasgow’s excess mortality (7). In another study, Walsh *et al*. (2015, 393) compared optimism and health in the three cities. They found no evidence that Glasgow’s population has lower levels of optimism, concluding that difference in psychological outlook is again an unlikely explanation. The 2016 GCPH report identifying the unknown factors assesses forty hypotheses, from rainfall and vitamin D deficiency, to sectarianism and genetics. Many hypotheses were unlikely or minimal, with the conclusion that Glasgow was more vulnerable to deindustrialisation and widening inequality since the 1970s. The press focus on lifestyle or colourful fringe hypotheses contrasts the public health reality.

### National identity and cultural mythology

Journalists often make arguments about Scottish national identity and cultural mythology while discussing the ‘Scottish effect’ and ‘Glasgow effect’. For example, the causes of poor public health are often attributed to a vague, stereotypical national character. In a 2009 *Guardian* article, Aida Edemariam and Kirsty Scott link heroin deaths to Scottishness while exploring the idea that heroin-related deaths of men under 45 could be a significant factor in Scotland’s excess mortality. The article cites an historian, a Leith city councillor and ex-heroin users in Edinburgh, yet Scottish author Irvine Welsh is quoted 10 times. Welsh is most famous for his 1993 novel *Trainspotting* about a group of charismatic Edinburgh heroin users in the late 1980s. The hugely successful film adaptation exported its lively but grim representation of Scotland worldwide. The *Guardian* article’s title refers to the ‘Trainspotting generation’ and the opening paragraph is a ninety-word quotation from *Trainspotting*’s famous ‘choose life’ monologue (Edemariam and Scott 2009, para 1 of 23). In the article, Welsh claims that heroin is used to cope with a devalued national identity, as ‘Englishness is the norm’ and Scottishness is ‘seen as a second-class thing’ (Edemariam and Scott 2009, para 11 of 23). Another risk factor is Scottish heroin users’ ‘distinct preference for needles’, which increases overdose risk compared with smoking. Welsh offers two reasons for this preference. First is the Scottish relationship to drugs: ‘It’s whisky vs beer […] In Scotland we’ve always gone for the dangerous hit. In England there’s always been a more mellow way—the slow pint of beer in a pub’ (para 13 of 23). Scottish drinking and drug use is reckless while the English example emphasises measured enjoyment, relaxation and sociality. Welsh’s second explanation is that ‘it’s more cost-effective to inject’ although he ‘[does not] want to stereotype’ (para 13 of 23). Cost-effectiveness is presumably a common concern, but Welsh implies that Scottish users inject because of stereotypical Scottish miserliness.

Welsh’s claims are highly dubious yet his voice and his works of fiction dominate the serious discussion of heroin-related deaths in this respected broadsheet. Prominent cultural figures like Welsh are used as key sources in public health discussions in the press and actively create the imaginary of an unhealthy Scottish character. Welsh resembles the figure of the black public intellectual. Political scientist Adolph Reed (2000, 83) critiques these self-elected spokespersons for black Americans: they position themselves ‘metaphorically at the boundary of the black experience’ but facing in, with enough distance for ‘group self-examination’. Yet their claims are often abstract or common sense because ‘prominence of author counts more than weight of utterance’ (Reed 2000, 82). Often writing in urban working-class Scots, Welsh is seen as an authentic spokesperson for contemporary Scottishness, despite spending much of his adult life in Dublin, Miami and Chicago (‘Take a Video Tour of Trainspotting Author Irvine Welsh’s £1m Chicago Home’ Live 2018). Welsh’s credentials are simply being Scottish, the insider providing insight for outsiders. This is not to equate the actual experiences of black Americans and (white) working-class Scots but to critique forms of representation that are dubiously perceived as authentic and accurate.

Other articles argue that health changes will improve the state of the nation. A 2004 article in *The Herald* claims that Scotland should be ‘a vibrant, confident young democracy looking forward to a dynamic future’ (Herald 2004, para 9 of 11). However, ‘[d]eprived of a Scottish parliament for nearly [300] years’, it is preoccupied by the past, neglecting contemporary problems (para 3 of 11). The article attributes poor public health to stubbornly self-destructive lifestyle choices: ‘we seem hamstrung by a large segment of the population determined to eat, smoke and drink themselves into early graves’ (para 9 of 11). It then connects the ‘Scottish effect’ to a ‘culture of negativity’, stress, pessimism and a sense of lack of control over life (para 10 of 11). This is a common explanation. It is Carol Craig’s central thesis in *The Scots’ Crisis of Confidence* (Craig 2011), first published in 2003, a remarkably well-received book despite largely relying, in Iain Ferguson’s words, on ‘unsubstantiated assertion and anecdote’ (Ferguson 2010, 301). Even GCPH director Professor Carol Tannahill connects the ‘Glasgow effect’ to a ‘prevailing overall culture of less optimism’ in a 2007 *Herald* article (Puttick 2007, para 6 of 21). This is reminiscent of philosopher (West 1994, 27) claim that there is ‘a kind of collective clinical depression in significant pockets of black America’. The biggest threat to black Americans is nihilism rather than oppression and exploitation (23). West’s solution is not structural change but a ‘*politics of conversion*’, that is, cultural or psychological change (original emphasis) (29). There is, then, a wider tendency to attribute growing relative poverty and social immobility to cultural and psychological disfigurations rather than structural factors, and to prescribe cultural reformation, or a wilful change in attitude, as a solution. The *Herald* article proposes a remarkably similar solution. The ‘traditional collectivist mentality’ cannot ‘turn the supertanker of Scottish negativity’ (‘When we learn to leave tradition behind and strike out for the future…’ 2004, para 10 of 11). Instead, individuals must ‘swop [sic] defeatism, helplessness and dependency for ambition, confidence and self-help’ (para 11 of 11). This is appealingly simple and, it seems, a patriotic duty if Scotland is to be a successful nation. Individual outlook is the answer to the nation’s problems.

Many ‘Glasgow effect’ articles portray imagined Glasgow residents or characterise the city itself. In an otherwise nuanced *Guardian* article, Ali Muriel depicts grotesque figures in Glasgow’s east end: ‘Pale men cluster outside windowless pubs puffing on cigarettes. A frail couple, three crutches between them, totter out of an off-licence […] An obese man with a withered leg’ limps while eating pizza (Muriel 2012, para 5 of 17). These figures are introduced by a quote from a local GP who claims ‘[y]ou don't need to be a doctor to see how unhealthy people in these communities are’ (para 5 of 17). Easily identifiable through its physical manifestation, ill health is simplified and safely confined, literally marginalised to the city’s edges. Occasional articles subvert bleak health warnings and moralistic advice, such as Tom Shields’s light-hearted piece on ‘man influenza’ in *The Herald* (Shields 2010, para 4 of 12). Noting that ‘[m]en with high testosterone levels are at greater risk’ (Shields 2010, para 4 of 12), Shields jokes that the root of the ‘Glasgow effect’ may be that Glasgow men are simply ‘too sexy for [their] life expectancy’ (para 9 of 12), adding that ‘[w]ith all that sex going on, you’re bound to have the odd cigarette and a glass of wine’ (para 10 of 12). Glaswegians, it appears, are either grotesquely unhealthy or charmingly bacchanalian. This split personality makes Glasgow special. Catriona Stewart in *The Herald* describes Glasgow as a ‘city of opposites’, a ‘glossy city centre with ragged, impoverished boundaries’, with ‘world-class sporting venues and malnutrition, idealism and inequality’—contradictions surely common to many major cities (Stewart 2013, para 1 of 11). Famous only for its ‘mortality rates’, Glasgow is not conventionally desirable compared with Edinburgh, Sydney or Vancouver (para 3 of 11). Yet Stewart asserts Glasgow’s value, concluding: ‘Edinburgh you could introduce to your mother; Glasgow would be ripe for a really dirty affair. That’s the thing about Glasgow: you know it could hurt you but it’s irresistible all the same’ (para 11 of 11). Glasgow is not just a faceless post-industrial city. Respectable Edinburgh dulls next to its dangerous charisma. Both negative and positive depictions contribute to the city’s mythology. Indeed, this tension gives Glasgow its imagined winning personality.

It is striking how often discussions of the ‘Glasgow effect’ or ‘Scottish effect’ return to ideas of national identity or characteristics. The names of the terms themselves invite this to some extent. But could we imagine a health trend described as the ‘Welsh effect’, for example, which is blamed on, and proof of, a distinctive Welsh character? Why is the ‘Glasgow effect’ so resonant on this level? The idea of a ‘Glasgow effect’ or ‘Scottish effect’, whether good or bad, emphasises Scottish or Glaswegian exceptionalism and charisma. The terms help create a strong identity and give Glasgow and Scotland a place in the world. They are, in other words, myths. Sociologist David McCrone (2001, 79) defines a myth as a story that helps define a group or nation to themselves and others, such as the American Dream. Literary scholar Gerard Carruthers outlines several Scottish identity myths: the fighting Scot, the freedom-loving Scot, the primitive Scot, the puritanical Scot and the civilised Scot (Carruthers 2009, 2). I would add the pathological Scot, or the myth of Scottish pathology. Indeed, the idea of cultural pathology is a key myth of Scotland.

There is a longstanding, prominent association between Scottishness and psychological splitting or pathology in intellectual discourse. This myth describes a split Scottish psyche, psychological malformation and intellectual deficiency. It has been promulgated by some of the most prominent figures of the Scottish intelligentsia for over a century. In 1919, literary critic G. Gregory Smith (1919, 4) coined the influential term Caledonian antisyzygy to describe the ‘zigzag of contradictions’ that supposedly characterise Scottish literature and national character, asking ‘[d]oes any other man (the Scot) combine so strangely the severe and tender in his character[?]’ (Smith 1919, 20). Meanwhile, in *Scott and Scotland* (1936), author Edwin Muir (1936) famously claims that ‘Scotsmen feel in one language (Scots) and think in another (standard English)’. This is ‘proof that the Scottish consciousness is divided’ (Muir 1936, 21) and signifies ‘the lack of a whole mind’ (22). This, Muir argues, is the ‘curse of Scottish literature’ (22). Psychological fracture afflicts and limits Scottish culture.

Vaguely clinical psychological language and the idea of Scottish psychopathology is also used in arguments on both sides of the Scottish independence debate. Writing in 1977, political theorist Tom Nairn (2015), in his classic book *The Break-up of Britain: Crisis and Neo-nationalism*, characterises Scotland using a colourful range of pathologising metaphors: the Union of 1707 was for Scotland ‘political castration’ (106), the separation of nation and state creating a ‘split personality’ (135), as well as ‘developmental oddities’ or ‘malformations’ compared with ‘nationalist norms elsewhere’ (140). Similar language is used in pro-Union arguments. Scottish author Morrison (2013, para 17 of 19), writing in *The Guardian* in 2013, diagnoses Scotland with borderline personality disorder (BPD) and argues against independence: ‘If Scotland has BPD, then its therapist would argue that breaking from a relationship and dreaming of a perfect new future’ would be ‘very bad for the patient’. It would be deeply uncomfortable and much more obviously controversial if such writers, as Scots, described agitation for independence as a symptom of psychopathology in, say, Catalonia or Quebec, or described those cultures as psychologically malformed. Yet these characterisations draw on and contribute to the myth of Scottish cultural pathology—as does the ‘Glasgow effect’.

While the myth of the pathological Scot may sound negative, it provides a strong sense of identity. Myths about Scottishness, McCrone (2001) argues, are ‘ideological device[s]’ for distinguishing Scotland from England, a project increasingly important as the countries grow more similar. This is something of Freud’s ‘narcissism of minor differences’, which Anton Blok (1998, 39) defines as ‘the idea that identity lies in difference, and difference is asserted, reinforced, and defended against what is closest and represents the greatest threat’. These differences are especially pertinent in the context of debates around Scottish devolution and independence, over the late 20th and early 21st centuries. Specifically, the ‘Glasgow effect’ is a tragic national myth. Political scientist Michael Morden explains that tragic nationalist myths emphasise collective pain, inevitable catastrophe and feelings of injustice. They are often associated with national minorities facing assimilation or loss of political status. Morden’s example is Quebec but this could also be applied to Scotland (Morden 2016, 459). The ‘Glasgow effect’ captures this sense of injustice and impending catastrophe. The politicisation of the terms is not wholly unwarranted since Scotland’s excess mortality is the result of vulnerability to political forces. Yet, this politicised discussion focuses on national character and mythology, so that the Union or a vague sense of ‘Scottishness’ is to blame over contemporary policymaking or a genuine democratic deficit.

### The ‘Glasgow effect’ and the construction of expertise

Journalists, academics and artists mobilised the ‘Glasgow effect’ and ‘Scottish effect’ terms to serve various agendas. The terms are what Katherine Smith calls chameleonic ideas. Chameleonic ideas ‘travel between actors and across boundaries’ because of their ‘elasticity and transformability’ (Smith 2013, 192). Although using shared terminology, each actor reinterprets the idea according to their interests, so usage may vary remarkably (192). This flexibility helps chameleonic ideas survive but can also limit their influence (192). Chameleonic ideas may morph so thoroughly that they influence policy in ways unanticipated or undesired by their originators (175). This is the case with GCPH, who lost control of the ‘Glasgow effect’ and ‘Scottish effect’ terms. Although vague, the terms sound specific enough to give the impression of people connected in a common project. Purporting to know something about the ‘Glasgow effect’ can offer power, influence or a platform. At same time, the terms were not focused enough to drive lasting policy change in any particular direction.

The press, even the quality press, does not simply disseminate GCPH’s claims about excess mortality for lay audiences. Instead, newspapers do their own narrative work with the ‘Glasgow effect’, introducing ideas to the debate that were not explicitly invited by GCPH’s research. Stephen Hilgartner (1990) explains that, in the culturally dominant view of science popularisation, scientists develop knowledge and then popularisers, such as the media, disseminate simplified accounts. Any differences between genuine and popularised science are caused by ‘distortion’ or ‘degradation’. This view, Hilgartner argues, is greatly oversimplified. It is difficult to find a precise boundary between science and popularised knowledge (Hilgartner 1990, 524). Popularisation is instead ‘a matter of degree’ (Hilgartner 1990, 528). I find that it is not simply that scientists, experts and academics use the terms ‘Scottish effect’ and ‘Glasgow effect’ soberly while the media distorts them irresponsibility. There are examples of the press using the terms carefully and precisely. Conversely, there are striking examples of academics discussing the ‘Glasgow effect’ with latitude, such as Shaw’s evacuation argument.

‘Glasgow effect’ continues to circulate, incorrectly defined or undefined, in academic and policy work. In a 2020 academic article on Scotland’s high drug rates, Kieran Sweeney (2020, 562) writes that the deindustrialisation of the 1970s and 1980s created a ‘legacy of displacement and deprivation, which has been referred to as “The Glasgow Effect”’. The ‘Glasgow effect’ here represents an historical context rather than unexplained excess mortality. Sweeney cites a 2019 House of Commons Scottish Affairs Committee report titled ‘Problem Drug Use in Scotland’. This report reads: ‘the Scottish Drugs Forum noted that economic changes between the 1960s and 1990s resulted in “dispossession and social displacement”, the legacy of which continues to manifest itself as “The Glasgow Effect” today’ (House of Commons Scottish Affairs Committee 2019, 13). The term is not defined or used again the report. The written evidence submitted by the Scottish Drug Forum defines the ‘Glasgow effect’ as ‘unexplained low life expectancy caused by premature deaths in children and younger adults’ (Scottish Drugs Forum 2019, 5). Glasgow Centre for Population Health (2010, 8), however, highlight that excess mortality is seen across all ages except children. These publications do not cite GCPH. ‘Glasgow effect’ still has currency, durability and flexibility in a public health context. This benefits the researchers and policy influencers adopting and adapting the term to serve their own agendas.

It is striking that ‘Glasgow effect’ is so loosely defined, even in academic and policy work. The line between the ‘Glasgow effect’ as a category of practice and category of analysis is problematically blurred. Sociologist Rogers Brubaker and historian Frederick Cooper define categories of practice as lay concepts used by ordinary people in everyday contexts; categories of analysis are used by analysts or scholars (Brubaker and Cooper 2000, 4)Brubaker and Cooper 2Brubaker and Cooper 200000Brubaker and Cooper 20000, 4. They warn against carelessly conflating lay and scholarly meanings as analytical concepts must be clearly defined to be effective. For example, ‘identity’ as a concept holds multiple meanings. As a category of practice, it may be used by individuals to understand themselves in relation to others, and by political actors to persuade people to understand themselves in particular ways (Brubaker and Cooper 2000). However, in academic work, ‘identity’ is used to stress fundamental sameness between people or in an individual over time, but also to highlight the fragmented instability of the self (Brubaker and Cooper 2000). These contradictory usages make the term too ambiguous for an effective analytical concept (Brubaker and Cooper 2000). ‘Glasgow effect’ is similarly ambiguous. As a category of practice, it accumulated multiple public health and cultural meanings. This is partly why GCPH retired it for the analytically sharper ‘excess mortality’. Yet ‘Glasgow effect’ continues to circulate as a category of analysis, used uncritically in academic and policy work. It may now be challenging to eradicate this usage.

GCPH’s original message about the existence of the ‘Glasgow effect’ was impactful yet its updated findings and retirement of the term received little recognition. The term has gained a sort of freestanding currency in certain circles. The vaccine-critical movement is a helpful comparison. As sociolegal scholar Anna Kirkland explains, vaccine critics brought academic, legal and political attention to the question of whether vaccines cause autism. Their arguments depended on the language of science but scientific consensus then disproved a vaccine-autism connection (Kirkland 2012, 70). Instead of accepting this, vaccine critics created an ‘alternative world of internal legitimacy’ mimicking the mainstream research world, including specialist journals and conferences (Kirkland 2012, 88). This, however, undercuts the wider external legitimacy of these groups (Kirkland 2012, 75). Ideas about health, especially when politicised, can take a life of their own and become self-sufficient. A loosely connected group of actors still use the ‘Glasgow effect’ concept. This group is like a legitimised version of the largely discredited vaccine-critical movement. It forms a self-referential world, with some continued legitimacy in policy circles. This works because of the role of experts in risk society. Helen Wells explains that since many risk issues are not easily observable to laypeople, experts who can identify and intervene in such processes are heavily relied on in policy decisions. Yet, since risk relates to chance and probability, there can be no single ‘über-expert’ on risk. This creates an ‘expert marketplace’ where multiple voices compete for authority (Wells 2011, 227). The researchers and others who adapt ‘Glasgow effect’ for their own agendas are competing to influence discussions about Scottish public health and validate their own expertise.

The example of the vaccine-critical movement also shows how new information can be resisted when it challenges an established idea. Thinking of the ‘Glasgow effect’ as a rumour or myth might explain why the concept persists. As psychologist Ralph L explains, rumours arise alongside anxieties and uncertainties (Rosnow 1988, 23). This is why facts alone cannot debunk a rumour that continues to cause anxiety; anxiety must be reduced before a rumour can effectively be refuted (24). Glasgow’s unexplained excess mortality was alarming and unclear, providing the ideal conditions for a rumour to spread and be sustained. According to political scientist Adam J Berinsky (2017, 245), people more readily accept rumours consistent with their pre-existing attitudes. Stereotypes of Scottish heavy drinking, drug use, depression, psychological split, negativity and poor diet may give extra credibility to the idea that cultural factors, or Scottishness itself, may cause Scotland’s excess mortality. Misinformation also persists due to the ‘continued influence effect’. Ullrich K H explains that misinformation, information initially believed valid but later retracted or corrected, continues to impact people’s memory and inferential reasoning, even after clear retractions and when individuals accurately remember these retractions (Ecker *et al*. 2014, 292). Psychologists O’Rear and Radvansky (2020, 141) similarly find in their experiments that most participants did not accept a retraction, even from authority figures. The ‘continued influence effect’ might explain why GCPH’s retraction of the term ‘Glasgow effect’, and its updated research on Glasgow’s excess mortality, has had limited impact. This myth is now unlikely to be neutralised through fact and refutation alone.

## Conclusion

Seven years after retiring the term, GCPH researchers are still contending with the ‘Glasgow effect’. A 2021 research article co-authored by GCPH’s David Walsh stresses that ‘there is no such thing as a ‘Glasgow effect’: rather it is a political effect and therefore requires a political response’ (Schofield *et al*. 2021, 67). GCPH may consider the mystery solved but the ‘Glasgow effect’ is firmly culturally embedded, continuing to circulate and doing its own work. The terms ‘Scottish effect’ and ‘Glasgow effect’ are ‘chameleonic ideas’ (Smith 2013). They are deployed in service of various agendas, while appearing to offer a common cause. Their vagueness and flexibility is exploited by a range of actors, including academics, journalists and cultural entrepreneurs, competing to influence policy and public opinion while establishing their own expertise. The ‘Glasgow effect’ especially is a mobile, flexible term, referring to poor public health and top athletic achievement, to a sick and an artistically rich culture. Beyond public health, ‘Scottish effect’ touches on Conservative decline in Scotland, Scotland’s influence on UK general elections, and Scottish independence. I do not think these resonances are deliberate or self-conscious but as a literary scholar I do find them satisfying. After all, Glasgow’s excess mortality can be attributed to a legacy of deindustrialisation, rapid cultural change and widening inequalities as a result of policies mostly under Thatcher’s government. This is a key context to Scotland’s push for devolution and independence. Indeed, the terms gained currency during the post-devolution and pre-independence referendum context. They resonated at a time when Scotland felt increased cultural confidence and political autonomy as well as increased anxiety over assimilation within the UK. This crossroads provoked a sense that the individual must take responsibility for their own health to protect and renew the nation. This myth, then, is not simply an extension of the larger myth of Scottish pathology, despite overlapping with and growing out of this. The ‘Glasgow effect’, the myth of a culture uniquely sick and uniquely artistic, became popular amidst renewed optimism and anxiety about Scotland’s future.

The example of the ‘Glasgow effect’ shows how health concepts can become politicised and wrapped in larger national or regional narratives. This raises further questions for future work. What other competing narratives and myths about Scottish health exist, alongside, before or since? Is there the equivalent of the ‘Glasgow effect’ in other countries or regions? What other health conditions are bound up with national concerns in different times or places? This study’s limited focus on newspapers also raises questions about readership and reception: how did readers encountering these terms in the Scottish press receive these myths and ideas, and did this shape their own understanding of health in terms of national identity? Further work could also consider how politicians and policy-makers handled these circling ideas around the ‘Glasgow effect’ and whether similar arguments were taken up in political rhetoric and public health messaging. To conclude, I want to ask whether public health researchers were justified in introducing such loaded terms as a way to raise awareness. Did the terms attract enough valuable research interest and funding to justify their use? This is difficult to gauge. However, given the persistence of rumour and myth, and the continued influence effect, I would argue that public health bodies need to be extremely careful about how they initially present research or hypotheses. GCPH perhaps realised too late that it could not control the usage of the ‘Glasgow effect’. The story of the ‘Glasgow effect’ serves as a cautionary tale for public health communicators, highlighting the difficulties of introducing complex and emerging public health ideas into a dynamic landscape of lay beliefs. However, the pre-existing meanings of ‘Scottish effect’ and ‘Glasgow effect’ meant GCPH never could have had full ownership of these terms. The continuing usage of ‘Glasgow effect’ in particular, in the press, academia and policy, suggests we are some way off seeing it fall out of circulation for good.

## Data Availability

No data are available.
